# Gray matter volumes discriminate cognitively impaired and unimpaired people with HIV

**DOI:** 10.1016/j.nicl.2021.102775

**Published:** 2021-07-31

**Authors:** Mikki Schantell, Brittany K. Taylor, Brandon J. Lew, Jennifer L. O'Neill, Pamela E. May, Susan Swindells, Tony W. Wilson

**Affiliations:** a Institute for Human Neuroscience, Boys Town National Research Hospital, Boys Town, NE, USA; b College of Medicine, University of Nebraska Medical Center (UNMC), Omaha, NE, USA; c Department of Internal Medicine, Division of Infectious Diseases, UNMC, Omaha, NE, USA; d Department of Neurological Sciences, UNMC, Omaha, NE, USA

**Keywords:** neuroHIV, HAND, Classification, Sex differences, Structural MRI

## Abstract

**Background:**

Current diagnostic criteria of HIV-associated neurocognitive disorders (HAND) rely on neuropsychological assessments. The aim of this study was to evaluate if gray matter volumes (GMV) can distinguish people with HAND, neurocognitively unimpaired people with HIV (unimpaired PWH), and uninfected controls using linear discriminant analyses.

**Methods:**

A total of 231 participants, including 110 PWH and 121 uninfected controls, completed a neuropsychological assessment and an MRI protocol. Among PWH, HAND (*n* = 48) and unimpaired PWH (*n* = 62) designations were determined using the widely accepted Frascati criteria. We then assessed the extent to which GMV, corrected for intracranial volume, could accurately distinguish the three groups using linear discriminant analysis. Sensitivity, specificity, positive likelihood ratio, negative likelihood ratio, area under the curve (AUC), and accuracy were computed for each model using the classification results based on GMV compared to the neuropsychological assessment.

**Results:**

The best performing model was comprised of bilaterally combined GMV and was stratified by sex. Among males, sensitivity was 85.2% (95% CI: 66.3%–95.8%), specificity was 97.0% (95% CI: 91.6%-99.4%), and the AUC was 0.91 (95% CI: 0.83–0.99). Among females, sensitivity was 100.0% (95% CI: 83.9%–100.0%), specificity was 98.8% (95% CI: 93.4%-100.0%), and the AUC was 0.99 (95% CI: 0.98–1.00).

**Conclusions:**

GMV accurately discriminated HAND from unimpaired PWH and controls. Measures of GMV may be highly sensitive to HAND, and revisions to the Frascati criteria should consider including GMV in conjunction with a neuropsychological assessment to diagnose HAND.

## Highlights

• HIV-associated neurocognitive disorders (HAND) are common in people with HIV.
• HAND is typically diagnosed using a neuropsychological assessment battery.
• We examined whether gray matter volumes can be used to identify HAND cases.
• Gray matter volumes accurately discriminated people with and without HAND.
• Stratifying by sex improved discriminability, revealing sex differences in HAND.

## 1 Introduction

Despite advances in combined antiretroviral therapy (cART), HIV-associated neurocognitive disorders (HAND) remain prevalent in about 40% of people with HIV (PWH; Masters and Ances, 2014). HAND appears to be even more prevalent in females with HIV, though these differences in cognitive function are often subtle and challenging to disentangle, especially because most HIV study cohorts are heavily biased towards males over females (Maki et al., 2015, Vance et al., 2017). The Frascati criteria (Antinori et al., 2007) are the most widely used research standard for classifying HAND and involve a neuropsychological assessment and self-reported measures of activities of daily living (ADLs). The Frascati criteria have been enormously beneficial in operationalizing a research framework for HAND, and because they stress sensitivity over specificity, the criteria are useful in detecting neurocognitive impairment before symptom onset (Tierney et al., 2017). However, the Frascati criteria’s HAND classifications have also been criticized for being imprecise and susceptible to biases (e.g., variability in test selection, differences in the number and types of cognitive domains assessed, and inconsistencies in impairment threshold definitions, thus increasing the false-positive frequency; Wang et al., 2019, Su et al., 2015, Meyer et al., 2013, Clifford and Ances, 2013).

Identifying predictive biomarkers of HAND and understanding sex-related differences in HIV infection is essential to improve diagnostic accuracy, responses to treatment, and advancing the field’s understanding of the pathophysiology of the disease (Rosenthal and Tyor, 2019). Neuropsychological assessments have been associated with structural magnetic resonance imaging (MRI) findings such as gray matter volumes (GMV) in HAND (Masters and Ances, 2014, Kato et al., 2020), though it is unclear whether GMV has the specificity to discriminate those with and without HAND. Thus, the primary goal of this study was to evaluate if GMV could accurately discriminate HAND, unimpaired PWH, and uninfected controls. Additionally, we assessed the utility of GMV as a diagnostic biomarker of HAND using neuropsychological assessment as the gold standard. We hypothesized that GMV would accurately classify individuals into HAND, unimpaired PWH, and uninfected control subgroups using linear discriminant analyses (LDA), and that the sensitivity and specificity of GMV would be moderate to excellent. We also predicted HAND would be more prevalent in females than in males in our sample, and because of this, there would be better discriminability of HAND among females compared to males.

## 2 Material and methods

### 2.1 Setting and participants

**Fig. 1:**
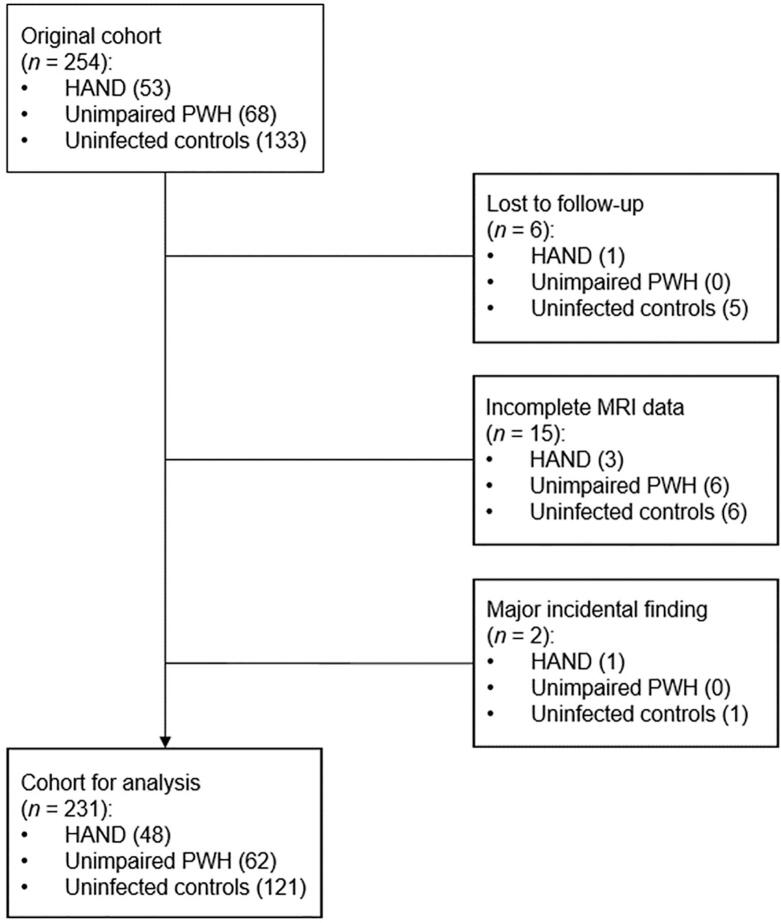
**Flow diagram of participant exclusions and final sample sizes.** Of the original 254 participants enrolled, 23 participants were excluded for missing or unusable MRI data and major incidental findings that could confound the results of the study.

Participants with HIV were recruited from the University of Nebraska Medical Center’s HIV Clinic, and uninfected controls were recruited from the Omaha area using a convenience sampling method. PWH were required to be on a cART regimen consistent with the current United States Department of Health and Human Services (DHHS) Guidelines for the Use of Antiretroviral Agents in Adults and Adolescents Living with HIV (DHHS, 2019), and to have an HIV-1 RNA viral load of < 50 copies/mL within three months of participation in the study. All controls were confirmed seronegative using the OraQuick *ADVANCE*® Rapid HIV-1/2 Antibody Test at the time of neuropsychological testing. Exclusion criteria included any neurological or psychiatric disorder (other than HAND), any chronic medical illness affecting CNS function (other than HIV), history of head trauma, current pregnancy, current substance use disorder, or ferrous metallic implants contraindicated for MRI (Fig. 1).

### 2.2 Standard protocol approvals, registrations, and patient consents

The University of Nebraska Medical Center’s Institutional Review Board reviewed and approved this protocol. All participants gave written informed consent following detailed description of the study.

### 2.3 Neuropsychological assessment

All participants underwent a neuropsychological assessment designed to assess HAND in accordance with the Frascati criteria (Antinori et al., 2007). The test battery assessed the following cognitive domains: *learning* (Hopkins Verbal Learning Test – Revised [HVLT-R] Learning Trials 1–3; Benedict et al., 1998), *memory* (HVLT-R Delayed Recall and Recognition Discriminability Index; Benedict et al., 1998), *executive functioning* (phonemic verbal fluency, semantic verbal fluency, Comalli Stroop Test Interference Trial, Trail Making Test Part B; Comalli et al., 1962, Heaton et al., 2004), *processing speed* (Trail Making Test Part A, Wechsler Adult Intelligence Scale, Third Edition [WAIS-III] Digit Symbol Coding, Comalli Stroop Test Color Trial; Comalli et al., 1962, Heaton et al., 2004, Wechsler, 1997), *attention* (WAIS-III Symbol Search, Comalli Stroop Test Word Trial Comalli et al., 1962, Heaton et al., 2004, Wechsler, 1997), and *motor* (Grooved Pegboard – Dominant and Non-Dominant Hands; Heaton et al., 2004, Kløve, 1963). We also assessed for *premorbid function* using the Wide-Range Achievement Test, Version 4 (WRAT-4) Word Reading Test (Wilkinson and Robertson, 2006).

Demographically corrected scores were obtained using published normative data (Comalli et al., 1962, Heaton et al., 2004, Wechsler, 1997, Kløve, 1963, Wilkinson and Robertson, 2006) and were computed to z-scores. Domain composite scores were computed by averaging the z-scores of assessments that comprised each respective cognitive domain. HAND classifications were assigned per the Frascati criteria (Antinori et al., 2007) by a neuropsychologist using the composite domain z-scores corrected for age, sex, race, and ethnicity, along with a modified version of the Lawton and Brody (1969) ADL scale to assess perceived functional impairment.

### 2.4 MRI data acquisition

Participant MRI data were acquired using an eight-channel head coil. Structural T1-weighted images were collected using a 3D-fast-field echo sequence on a Philips Achieva 3.0 T X-Series scanner. The parameters for the 3D-fast-field echo sequence were as follows: TR: 8.09 ms; TE: 3.7 ms; field of view: 24 cm; matrix: 256 × 256; slice thickness: 1 mm with no gap; in-plane resolution: 0.9375 × 0.9375 mm; sense factor: 1.5. The anatomical images were examined by a radiologist for incidental findings.

### 2.5 Structural MRI data processing of GMV

**Fig. 2:**
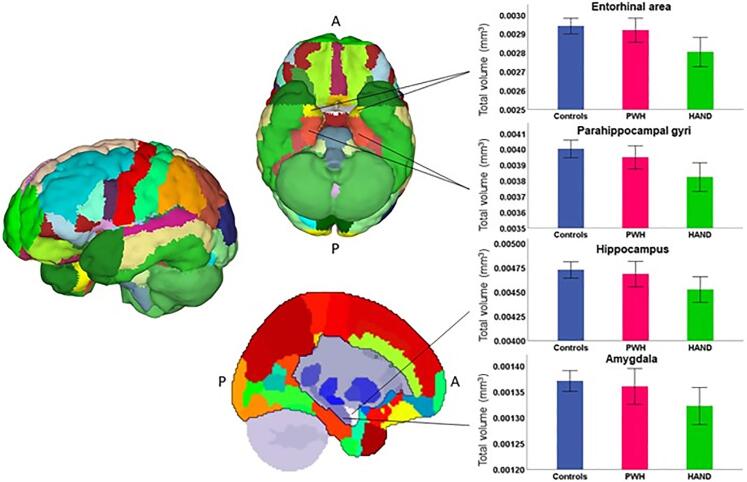
**Boxplots showing exemplary groupwise distributions of regions using the Neuromorphometrics atlas.** Volumes (in mm^3^) were summed bilaterally and corrected for intracranial volume (ICV). Those with HAND consistently had less GMV compared to unimpaired PWH and controls. Error bars display 95% confidence intervals.

The T1-weighted anatomical images were segmented into gray matter, white matter, and cerebrospinal fluid using the standard voxel-based morphometry (VBM) approach in the computational anatomy toolbox (CAT12 v12.6; Gaser and Dahnke, 2016) in Statistical Parametric Mapping (SPM12) software. The acquired T1-weighted images were noise reduced using a spatially-adaptive non-local means (SANLM) denoising filter (Manjón et al., 2010) and a classic Markov Random Field approach (Rajapakse et al., 1997). The images were then bias corrected using an affine registration and a local intensity transformation. Additionally, the images were segmented using an adaptive maximum a posteriori technique (Ashburner and Friston, 2005) and a partial volume estimation with a simplified mixed model of two tissue types or less (Tohka et al., 2004). Finally, the images were normalized to MNI space and smoothed using an 8 mm full-width at half maximum (FWHM) Gaussian kernel. The Neuromorphometrics atlas (Caviness et al., 1999) was then applied to determine gray matter volumes within the regions comprising the atlas (Fig. 2). Groupwise distributions of GMV were derived using the Neuromorphometrics Scalable Brain Atlas (Bakker et al., 2015) and the Three-Dimensional Brain Atlas Reconstructor (3dBAR; Majka et al., 2013).

### 2.6 Statistical analyses

Group differences in demographic and neuropsychological variables were assessed using one-way ANOVAs and *χ^2^* tests. One-way ANOVAs were also used to assess for differences in HIV metrics such as years since HIV diagnosis, years on cART, CD4 nadir, and current CD4 counts among unimpaired PWH and HAND by sex.

To examine the discriminability of GMV among those with HAND, unimpaired PWH, and uninfected controls, we used linear discriminant analyses in which classification was computed from group sizes. Because we had a total of three groups, two discriminant functions were returned from each linear discriminant analysis. All explanatory variables of interest were assessed for normality, multicollinearity, and outliers by examining skewness and kurtosis, and using pooled covariance matrices across all groups together and between groups. Bilateral GMV were summed together per region to reduce collinearity and were corrected for total intracranial volume (ICV) per person to remove the confounding effects of total brain size. This yielded a total of 61 bilateral cortical and subcortical gray matter regions, each corrected for ICV, which were included as explanatory variables in the linear discriminant analysis with group (HAND, unimpaired PWH, and uninfected control) as the response variable. In a subsequent analysis, we stratified the model by sex to assess differences in discriminability between males and females.

Sensitivity, specificity, positive likelihood ratio (LR + ), negative likelihood ratio (LR-), and area under the curve (AUC) were calculated using the classification results from the linear discriminant analyses. We used LR + and LR- to determine diagnostic accuracy over positive and negative predictive values because these metrics are more generalizable to other study populations (Fischer et al., 2003). We used leave-one-out cross-validation analyses to account for possible over-fitting. All statistical analyses were conducted in IBM SPSS Statistics v.26.

### 2.7 Data availability policy

Requests for data can be fulfilled via the corresponding author. Upon completion of the study, data will be made available to the public through the National NeuroAIDS Tissue Consortium (NNTC) database.

## 3 Results

### 3.1 Participants

Of the 254 participants recruited for the study, 231 participants (110 PWH [62 unimpaired PWH and 48 HAND] and 121 controls) successfully completed the neuropsychological battery and the MRI (Fig. 1). Participants who were lost to follow-up (*n* = 6), had incomplete MRI data (*n* = 15), or had major incidental findings such as a brain tumor (*n* = 2) were excluded from the analyses.

### 3.2 Descriptive data

The three groups stratified by sex had comparable demographic characteristics (Table 1). Thus, standardized values were used for the primary MRI-based analyses. HIV-related measures such as years since HIV diagnosis, years on cART, nadir CD4 counts, and current CD4 counts were similar between the unimpaired PWH and HAND groups (Table 1 and Supplementary Table e-1). All PWH were virally suppressed (HIV viral load < 50 copies/mL) as part of the inclusion criteria.

**Table 1:** **Participant demographics and neuropsychological profiles.** Domain scores were calculated by averaging individual assessment z-scores in each respective domain. Values in the parentheses are standard deviations (SD). HAND – HIV-associated neurocognitive disorder, PWH – People with HIV, ART – Antiretroviral therapy, SD – Standard deviation. ^a^ χ^2^ test. ^b^ Eight participants (2 HAND, 3 unimpaired PWH, and 3 uninfected controls) could not complete the task.

	HAND (*n* = 48)		Unimpaired PWH (*n* = 62)		Uninfected Controls (*n* = 121)		*p*-value
	Males (*n* = 27)	Females (*n* = 21)	Males (*n* = 37)	Females (*n* = 25)	Males (*n* = 64)	Females (*n* = 57)	
Mean age (SD)	44.07 (12.60)	50.29 (10.66)	48.05 (14.09)	47.04 (9.97)	43.34 (15.83)	44.86 (15.05)	0.324
Race (frequency, %)							
White	17 (63%)	9 (43%)	29 (78%)	16 (64%)	46 (72%)	36 (63%)	0.113^a^
Non-White	10 (37%)	12 (57%)	8 (22%)	9 (36%)	18 (28%)	21 (37%)	
Mean years since HIV diagnosis (SD)	11.48 (7.73)	12.05 (7.41)	11.27 (7.58)	9.92 (6.70)	–	–	0.786
Mean years on ART (SD)	10.33 (7.05)	9.29 (6.70)	9.19 (6.47)	7.98 (6.40)	–	–	0.648
Mean CD4 nadir (cells/µL, SD)	216.59 (153.81)	230.48 (169.59)	256.50 (159.34)	251.36 (180.15)	–	–	0.779
Mean current CD4 count (cells/µL, SD)	709.81 (414.56)	808.76 (405.82)	791.11 (446.81)	783.04 (444.41)	–	–	0.848
Mean learning domain z-score	−1.54 (1.08)	−1.72 (1.14)	−0.31 (0.98)	0.00 (0.62)	−0.733 (1.23)	−0.31 (1.07)	<0.001
Mean memory domain z-score	−1.11 (1.09)	−1.11 (0.90)	−0.08 (0.74)	0.05 (0.57)	−0.43 (1.09)	−0.13 (0.81)	<0.001
Mean motor domain z-score	−0.93 (0.82)	−1.15 (0.94)	−0.28 (1.00)	−0.18 (1.06)	−0.44 (0.84)	−0.19 (1.04)	<0.001^b^
Mean attention domain z-score	−0.91 (0.89)	−1.07 (0.80)	0.05 (0.60)	−0.01 (0.56)	0.23 (0.88)	0.03 (0.87)	<0.001
Mean processing speed domain z-score	−0.68 (0.59)	−0.54 (0.73)	0.31 (0.74)	0.15 (0.73)	0.15 (0.64)	0.13 (0.83)	<0.001
Mean executive function domain z-score	−0.77 (0.59)	−0.65 (0.78)	0.13 (0.51)	0.05 (0.57)	0.01 (0.68)	−0.13 (0.81)	<0.001

### 3.3 Neuropsychological testing results

Of the 110 PWH, 48 (43.6%) were classified as having HAND using the neuropsychological and functional assessment as the gold standard. Among those classified as HAND, 39 (81.3%) were classified as having asymptomatic neurocognitive impairment (ANI), 5 (10.4%) were classified as having mild neurocognitive disorder (MND), and 4 (8.3%) were classified as having HIV-associated dementia (HAD). Among the controls, 18 (14.9%) scored at least one SD below the mean on two or more domains, and thus were cognitively impaired based on the Frascati criteria. The remaining 103 (85.1%) controls scored within the normative range or higher on the neuropsychological assessments.

Groupwise comparisons of each domain showed statistically significant differences in the motor, learning, memory, executive function, processing speed, and attention domains (*p* < 0.001). Post hoc Tukey HSD tests showed that male and female HAND participants consistently performed worse on all neuropsychological domains compared to the other two groups (*p* < 0.001), while unimpaired PWH and controls performed similarly (*p* > 0.05; Table 1 and Supplementary Table e-1).

### 3.4 Gray matter volume results

GMV was measured across a total of 54 cortical and 7 subcortical regions using the Neuromorphometrics atlas (Caviness et al., 1999). PWH, including HAND and unimpaired PWH, showed widespread reductions in GMV relative to controls (Fig. 2; Supplementary Table e-2).

### 3.5 Linear discriminant analyses

**Fig. 3:**
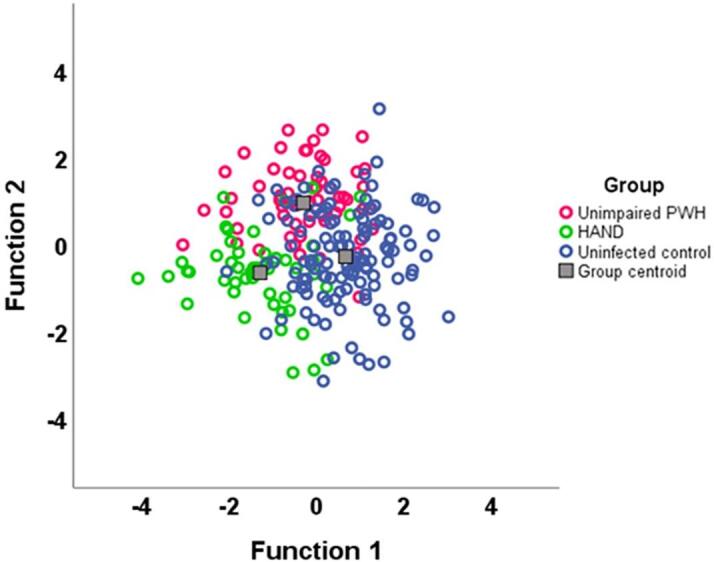
**Scatterplot displaying group clustering.** The clustering was based on two canonical discriminant functions using the bilaterally summed GMV values corrected for ICV. Function 1 discriminated HAND and controls, while Function 2 discriminated HAND and unimpaired PWH.

Two linear discriminant analyses were conducted to classify those with HAND, unimpaired PWH, and controls based on GMV. The first linear discriminant analysis utilized the GMV measured in each of the 61 regions (54 cortical, 7 subcortical), which were summed bilaterally and corrected for intracranial volume. Classification was based on group sizes, with the prior probabilities of being classified into each group as follows: HAND = 0.208, unimpaired PWH = 0.268, and controls = 0.524. The model returned two discriminant functions that combined accounted for 54.9% of the variance, λ = 0.45, *p* = 0.017 (Fig. 3).

The first discriminant function alone, which discriminated HAND from controls, accounted for 37.8% of the variance in group membership. The second discriminant function, which discriminated HAND from unimpaired PWH, accounted for the remaining variance. The sensitivity was 70.8% (95% CI: 55.9% − 83.1%), and the specificity was 92.9% (95% CI: 88.2% − 96.2%; Table 2). The regions that contributed the most to the model based on the size of the absolute values (i.e., coefficients with higher absolute values correspond to variables with greater discriminability) can be derived through the standardized canonical discriminant function coefficients in Supplementary Table e-3. For the confusion matrix of the full model, refer to Supplementary Table e-4.

**Table 2:** **Test diagnostics of GMV compared to a neuropsychological and functional assessment as the gold standard.** Sensitivity, specificity, LR+, LR-, AUC, and accuracy were computed using the classification results from the linear discriminant analyses. Regions were summed bilaterally and corrected for ICV. LR+ - Positive Likelihood Ratio, LR- – Negative Likelihood Ratio, CI – Confidence Interval, AUC – Area Under the Curve.

	Sensitivity (95% CI)	Specificity (95% CI)	LR+ (95% CI)	LR- (95% CI)	AUC (95% CI)	Accuracy (95% CI)
Combined Sample	70.8%	92.9%	9.97	0.31	0.82	71.9% (66.1%-77.7%)
	(55.9%-83.1%)	(88.2%-96.2%)	(5.73–17.36)	(0.20–0.49)	(0.74–0.90)	
Males Only	85.2%	97.0%	28.68	0.15	0.91	87.5% (81.8%-93.2%)
	(66.3%-95.8%)	(91.6%-99.4%)	(9.30–88.40)	(0.06–0.38)	(0.83–0.99)	
Females Only	100.0%	98.8%	82.00	0.00	0.99	96.1% (92.4%-99.8%)
	(83.9%-100.0%)	(93.4%-100.0%)	(11.69–575.21)	(0.00–0.00)	(0.98–1.00)	
Overall Model Stratified by Sex	91.7%	97.8%	41.94	0.09	0.95	91.3% (87.7%-95.0%)
	(80.0%-97.7%)	(94.5%-99.4%)	(15.85–110.96)	(0.03–0.22)	(0.90–0.99)	

We then stratified the model by sex, and two discriminant functions were returned for both the males-only and females-only models. Among males, the prior probabilities for each group were: HAND = 0.211, unimpaired PWH = 0.289, and controls = 0.500. The two discriminant functions among males accounted for 77.4% of the variance, λ = 0.226, *p* = 0.111. The first discriminant function alone, which discriminated HAND from controls, accounted for 61.0% of the variance in group membership. The second discriminant function, which discriminated HAND and unimpaired PWH, accounted for the remaining variance. Among males, the sensitivity was 85.2% (95% CI: 66.3–95.8%), and the specificity was 97.0% (95% CI: 91.6–99.4%). See Supplementary Table e-5 for the confusion matrix of the male model.

**Fig. 4:**
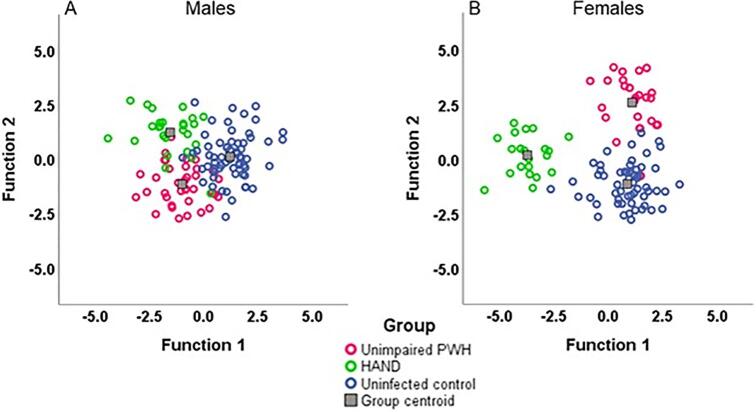
**Scatterplots displaying the two canonical discriminant functions stratified by sex.** (A) shows group clustering based on the two discriminant functions among males using bilaterally summed GMV values corrected for ICV. Function 1 discriminates HAND and controls, while Function 2 discriminates HAND and unimpaired PWH. (B) shows group clustering based on the two discriminant functions among females using bilaterally summed GMV values corrected for ICV. Function 1 discriminates HAND and unimpaired PWH, and Function 2 discriminates unimpaired PWH and controls.

Among females, prior probabilities of being classified into each group were HAND = 0.204, PWH = 0.243, and controls = 0.553. Two discriminant functions were returned, accounting for 93.7% of the variance, λ = 0.063, *p* < 0.001 (Fig. 4). The first discriminant function discriminated HAND from unimpaired PWH, and alone accounted for 78.5% of the variance in group membership. The second discriminant function, which discriminated unimpaired PWH and controls, accounted for the remaining variance. Among females, the sensitivity was 100.0% (95% CI: 83.9% − 100.0%), and the specificity was 98.8% (95% CI: 93.4% − 100.0%). For the confusion matrix of the female model, see Supplementary Table e-6. Overall, the sensitivity of the stratified model was 91.7% (95% CI: 80.0% − 97.7%), and the specificity was 97.8% (95% CI: 94.5% − 99.4%). Supplementary Table e-7 shows the confusion matrix for the overall model stratified by sex.

To account for over-fitting, leave-one-out cross-validation methods were used. In the full bilaterally combined model, 46.8% of the cross-validated grouped cases were classified correctly, 43.0% were classified correctly among males, and 54.4% were classified correctly among females. Classification of unimpaired PWH and HAND was better than chance based on the prior probabilities for all models, and classification of the controls was below chance.

## 4 Discussion

The model that balanced sensitivity and specificity the best was the bilaterally combined GMV model stratified by sex, specifically the females-only model. This model performed remarkably well with an AUC of 0.99 (95% CI: 0.98–1.00), a sensitivity of 100.0% (95% CI: 83.9%-100.0%), and a specificity of 98.8% (95% CI: 93.4%-100.0%).

Though the females-only model performed better than both the males-only model and the full model, it is not clear why this is. Previous studies have identified biological sex differences in HIV-related immune activation (e.g., increased CD8 + T cell activation and interferon-stimulated genes [Chang et al., 2013, Meier et al., 2009, Raghavan et al., 2017] in females relative to males), and chronic comorbidities such as increased cardiovascular (Triant et al., 2007) and cerebrovascular (Cruse et al., 2012) event risk in females with HIV, though it is not known how these differences relate to cognition, especially in relation to HAND (Cysique and Brew, 2019). Additionally, the role of health disparities should be examined further. It has been well-documented that women with HIV are more likely to be women of color, experience early life stress, encounter domestic and community violence later in life, live in poverty, face barriers to health care access, and ultimately have lower levels of educational attainment than men with HIV (Sundermann et al., 2018, Rubin et al., 2015, Rubin et al., 2017). This lower level of educational attainment and increase in traumatic stress and life adversity in females with HIV could potentially explain why females are disproportionately affected by HAND (Sundermann et al., 2018, Rubin et al., 2015, Rubin et al., 2017, Rubin et al., 2016). Though differences in important demographic variables such as race were not significantly different by sex and group in the present study, these differences were not trivial either and this should be kept in mind. Future research should focus on elucidating the underlying mechanisms for the higher HAND rate, with a particular focus on understanding the influence of biological sex.

The Frascati criteria have been criticized for overestimating impairment in PWH, and more specifically, overestimating the prevalence of asymptomatic neurocognitive impairment (Gisslén et al., 2011). This is because 16% of the population is expected to score more than one standard deviation below the mean on any given neuropsychological test (Gisslén et al., 2011). This could explain why the discriminant models using GMV had higher specificities than sensitivities. In other words, the GMV may have been correctly classifying HAND, but the Frascati criteria used to define HAND in this sample may have been too liberal (i.e., identified some individuals as having HAND who did not truly have HAND). This would lead to a high number of false positives, which can have serious implications in the lives of PWH. Given its high sensitivity, using the Frascati criteria as a screening tool in the context of serial testing procedures may be more ideal. For example, the Frascati criteria could be used initially and, if a participant screens positive for HAND, a more specific test could be conducted to prevent false positive HAND diagnoses. Potentially, the GMV algorithm tested in the present study could be used for this purpose, but further testing and confirmation studies are need. In addition, more analyses should be conducted with different diagnostic criteria such as the Gisslén criteria (Gisslén et al., 2011), the global deficit score (GDS; Carey et al., 2004), and the Diagnostic and Statistical Manual of Mental Disorders (DSM-5) criteria (Tierney et al., 2017, American Psychiatric Association, 2013, Underwood et al., 2018) to determine if the sensitivities of the GMV improve when HAND is not potentially overestimated.

Considering the small HAND sample in this study, it is hard to ascertain which brain regions were most important for discriminating HAND from unimpaired PWH and controls given the widespread reductions in GMV, which is consistent with the literature (Kato et al., 2020, Gisslén et al., 2011, Sanford et al., 2018a, Sanford et al., 2018b, Sanford et al., 2019, Guha et al., 2016). However, there was less GMV across many brain regions in those with HAND compared to controls and unimpaired PWH, including the inferior and middle temporal gyri, superior medial frontal gyri, amygdala, hippocampus, entorhinal cortices, fusiform gyri, posterior cingulate gyri, and the planum temporale. Interestingly, lower nadir CD4 levels have been associated with decreased subcortical GMV, and the greater the disparity between nadir CD4 and current normal CD4 counts, the worse the structural integrity of the brain (Jernigan et al., 2011, Martín-Bejarano García et al., 2021, Nir et al., 2021). Though we did not observe significant sex and group differences in nadir CD4 and current CD4 counts, females with HAND had a greater numerical disparity between nadir CD4 and current CD4 counts than males with HAND.

While GMV may be potentially useful clinically, more mechanistic analyses need to be done. Specifically, it is not clear what causes HAND, and there are many factors that have been identified as possibly contributing to the development of HAND. One potentially useful step might be to adopt neuroimaging markers of disease into the diagnostic criteria, much like how the National Institute on Aging and the Alzheimer’s Association has strived to move toward incorporating more biological measures into the framework for identifying Alzheimer’s disease (AD). These markers of AD currently include neurodegenerative metrics obtained from MRI (e.g., less GMV) and PET (e.g., abnormal amyloid and tau depositions; Jack et al., 2018). Potential future directions might be to combine the Frascati criteria with emerging neuroimaging markers of HAND, including GMV as demonstrated in the present study, as well as functional MRI (Ances et al., 2009, Nguchu et al., 2021, Plessis et al., 2014, Hall et al., 2021, Minosse et al., 2021) and magnetoencephalography (Spooner et al., 2020, Lew et al., 2018, Wiesman et al., 2018, Wilson et al., 2017., Becker et al., 2012a, Becker et al., 2012b). To this end, more specific phenotypes of cognitive impairment based on GMV and comparable neuroimaging metrics should be investigated and reproduced in other studies, specifically in cohort studies, and iteratively refined to gain a better clinical gestalt of HAND.

Before closing, it is important to recognize the limitations of this study. First, we used a cross-sectional design and future studies should consider a longitudinal approach. Second, the results from the GMV assessments would ideally be used together with the neuropsychological results to inform HAND diagnoses. However, because our groups were defined based on the results of the neuropsychological assessment, we did not explore the diagnostic accuracy of the GMV and neuropsychological results combined. Third, although HAND was moderately prevalent in our sample of PWH (43.6%), we were limited in our analyses due to the relatively small HAND sample (*n* = 48), and those with HAND has relatively mild cognitive impairment which limits the comparability of the neuropsychological and MRI-metric’s predictive utility. These factors along with other unmeasured factors could be contributing to the wide and relatively imprecise confidence intervals obtained in the analyses. Future work should conduct additional testing in larger scale studies to validate and refine the models developed in the present study. Fourth, we used a whole brain approach, which on one hand could result in overfitting of the model, but on the other may be more appropriate clinically and less biased. This approach can be largely automated and thus implemented in many clinical settings, which makes it advantageous in many ways. However, diagnostic MRI is expensive and may not be easily accessible for all PWH, specifically in resource-limited settings. Further, such automated analyses require all MRI data to be transformed into a standardized space (e.g., MNI space) during pre-processing. While this process has been heavily refined over the past 20 years and is very reliable, there is some loss of precision with the process and that should be kept in mind. Finally, though we tried to control for demographic variables across all groups, there was a higher percentage of females with HAND who were non-White compared to any other group. While this difference was not statistically significant, it was a non-trivial difference and reflects the nature of the HIV epidemic (Sundermann et al., 2018, Rubin et al., 2015, Rubin et al., 2017, Rubin et al., 2016).

While we sought to reduce bias whenever possible in this study, our results may not be generalizable to the entire population of PWH. In particular, the controls were recruited using a convenience sampling method, thereby limiting the generalizability of the results of the study and biasing the estimates derived from the sample. Additionally, patients with any neurological or psychiatric conditions, major chronic health comorbidities (e.g., cancer), and ferromagnetic implants were excluded, and PWH were also required to be virally suppressed, so the results of the study should be interpreted accordingly. Because of this, there is also a concern of spectrum bias, which is why it is essential to test these methods in other study samples.

## 5 Conclusion

These results show that GMV may be useful to aid in the identification of HAND and help clinicians better understand the disorder. Our results also suggest that incorporating more biologically-based measures into the framework for defining HAND could be of significant benefit. Specifically, our discriminant model stratified by sex had a sensitivity of 91.7% and a specificity of 97.8%, with the females-only model (sensitivity = 100.0%, specificity = 98.8%) discriminating HAND better than the males-only model (sensitivity = 85.2%, specificity = 97.0%). These findings warrant further investigation into the sex differences among those with HAND. To close, these findings provide compelling evidence that HAND can be detected using GMV and may be of major value to the diagnostic framework for identifying HAND in neuroHIV.

## CRediT authorship contribution statement

**Mikki Schantell:** Conceptualization, Data curation, Formal analysis, Investigation, Validation, Visualization, Writing – original draft, Writing – review & editing. **Brittany K. Taylor:** Formal analysis, Investigation, Validation, Writing - review & editing. **Brandon J. Lew:** Formal analysis, Investigation, Validation, Writing - review & editing. **Jennifer L. O’Neill:** Formal analysis. **Pamela E. May:** Formal analysis, Investigation, Validation, Writing - review & editing. **Susan Swindells:** Conceptualization, Data curation, Funding acquisition, Investigation, Methodology, Project administration, Supervision, Validation, Writing - review & editing. **Tony W. Wilson:** Conceptualization, Data curation, Funding acquisition, Investigation, Methodology, Project administration, Resources, Supervision, Validation, Writing - review & editing.

## Declaration of Competing Interest

The authors declare that they have no known competing financial interests or personal relationships that could have appeared to influence the work reported in this paper.
