# Supplementary material for: Gray matter volumes discriminate cognitively impaired and unimpaired people with HIV
Source: Neuroimage Clin. 2021 Jul 31;31:102775. doi: 10.1016/j.nicl.2021.102775 (PMC8358696; doi:10.1016/j.nicl.2021.102775)
Supplement: Supplementary data 1 [file mmc1.docx]

Supplementary Table e-1. Participant demographics and neuropsychological profiles by group and sex.

|  | HAND  (*n* = 48) | | Unimpaired PWH  (*n* = 62) | | Uninfected Controls  (*n* = 121) | | *p*-value |
| --- | --- | --- | --- | --- | --- | --- | --- |
|  | Males  (*n* = 27) | Females  (*n* = 21) | Males  (*n* = 37) | Females  (*n* = 25) | Males  (*n* = 64) | Females  (*n* = 57) |  |
| Median age (IQR) | 44 (33-54) | 52 (42-58) | 52 (35-59) | 49 (39-54) | 40 (28-58) | 45 (30 – 58) | 0.324 |
| Race (frequency, %) |  |  |  |  |  |  |  |
| White | 17 (63%) | 9 (43%) | 29 (78%) | 16 (64%) | 46 (72%) | 36 (63%) | 0.113^a^ |
| Non-White | 10 (37%) | 12 (57%) | 8 (22%) | 9 (36%) | 18 (28%) | 21 (37%) |  |
| Median years since HIV diagnosis (IQR) | 12 (4-19) | 9 (5-20) | 11 (5-15) | 9 (5-16) | - | - | 0.786 |
| Median years on ART (IQR) | 10 (3-17) | 9 (4-15) | 8 (3-15) | 7 (1-7) | - | - | 0.648 |
| Median CD4 nadir (cells/µL, IQR) | 195.00  (93.00-323.00) | 272.00  (47.00-403.50) | 240.50  (143.75-340.50) | 223.00  (96.00-386.00) | - | - | 0.779 |
| Median current CD4 count (cells/µL, IQR) | 632.00  (405.00-1028.00) | 741.00  (481.50-1089.00) | 714.00  (479.50-967.00) | 780.00  (395.00-985.00) | - | - | 0.848 |
| Median learning domain z-score (IQR) | -1.70  (-2.50 - -0.25) | -1.10  (-1.55 - -0.48) | -0.40  (-1.10-0.45) | 0.00  (-0.50 – 0.35) | -0.80  (-1.50-0.30) | -0.20  (-0.90-0.35) | <0.001 |
| Median memory domain z-score (IQR) | -0.85  (-2.05 - -0.60) | -2.20  (-2.65 - -0.60) | -0.40  (-1.10-0.45) | 0.00  (-0.50 – 0.35) | -0.80  (-1.50-0.30) | -0.20  (-0.90-0.35) | <0.001 |
| Median motor domain z-score (IQR) | -0.85  (-1.80 - -0.50) | -1.00  (-1.75 - -0.40) | -0.35  (-1.13-0.23) | -0.20  (-0.60-0.40) | -0.55  (-0.98 – 0.05) | -0.35  (-1.00-0.63) | <0.001 |
| Median attention domain z-score (IQR) | -0.69  (-1.48 - -0.31) | -1.08  (-1.53 - -0.50) | 0.21  (-0.36-0.39) | -0.14  (-0.37-0.49) | 0.35  (-0.24-0.72) | 0.04  (-0.38-0.60) | <0.001 |
| Median processing speed domain z-score (IQR) | -0.58  (-0.95 - -0.38) | -0.42  (-0.96-0.02) | 0.19  (-0.18-0.63) | -0.09  (-0.40-0.02) | 0.20  (-0.17-0.56) | 0.10  (-0.37-0.66) | <0.001 |
| Median executive function domain z-score (IQR) | -0.79  (-1.26 - -0.35) | -0.68  (-1.21 - -0.29) | 0.03  (-0.19-0.44) | 0.04  (-0.41-0.41) | 0.12  (-0.50-0.57) | -0.06  (-0.47-0.46) | <0.001 |

Domain scores were calculated by averaging individual assessment z-scores in each respective domain. HAND – HIV-associated neurocognitive disorder, PWH – People with HIV, ART – Antiretroviral therapy. IQR – Interquartile range.

^a^ *χ^2^* test.

Supplementary Table e-2. Exploratory analysis of group- and sex-wise differences.

|  | **HAND** | | | | **Unimpaired PWH** | | | | **Seronegative Controls** | | | | | |
| --- | --- | --- | --- | --- | --- | --- | --- | --- | --- | --- | --- | --- | --- | --- |
|  | **Males** | | **Females** | | **Males** | | **Females** | | **Males** | | **Females** | |  | |
| **Regions** | **Mean** | **SD** | **Mean** | **SD** | **Mean** | **SD** | **Mean** | **SD** | **Mean** | **SD** | **Mean** | **SD** | ***p*-value** |  |
|  |  |  |  |  |  |  |  |  |  |  |  |  |  |  |
| Accumbens | 0.000580 | 0.000090 | 0.000602 | 0.000078 | 0.000595 | 0.000075 | 0.000615 | 0.000059 | 0.000599 | 0.000064 | 0.000613 | 0.000069 | 0.409 |  |
| Amygdala | 0.001318 | 0.000120 | 0.001329 | 0.000130 | 0.001356 | 0.000136 | 0.001366 | 0.000140 | 0.001357 | 0.000113 | 0.001386 | 0.000109 | 0.196 |  |
| Caudate | 0.003921 | 0.000528 | 0.004352 | 0.000487 | 0.004090 | 0.000563 | 0.004323 | 0.000541 | 0.004097 | 0.000521 | 0.004245 | 0.000578 | 0.026 |  |
| Exterior Cerebellum | 0.051156 | 0.005489 | 0.053026 | 0.005348 | 0.049417 | 0.008524 | 0.052884 | 0.003876 | 0.051185 | 0.005967 | 0.052886 | 0.004385 | 0.058 |  |
| Hippocampus | 0.004407 | 0.000469 | 0.004675 | 0.000389 | 0.004502 | 0.000442 | 0.004952 | 0.000502 | 0.004652 | 0.000496 | 0.004810 | 0.000416 | 0.000* |  |
| Pallidum | 0.000323 | 0.000096 | 0.000388 | 0.000162 | 0.000344 | 0.000140 | 0.000380 | 0.000100 | 0.000351 | 0.000083 | 0.000381 | 0.000115 | 0.161 |  |
| Putamen | 0.005462 | 0.000960 | 0.005810 | 0.000563 | 0.005392 | 0.000680 | 0.005821 | 0.000676 | 0.005597 | 0.000561 | 0.005718 | 0.000694 | 0.064 |  |
| Thalamus | 0.006297 | 0.001110 | 0.007374 | 0.000826 | 0.006612 | 0.000826 | 0.007316 | 0.000896 | 0.006730 | 0.000658 | 0.007498 | 0.000727 | 0.000* |  |
| Cerebellar Vermal Lobules, I-V | 0.002376 | 0.000222 | 0.002413 | 0.000281 | 0.002337 | 0.000318 | 0.002494 | 0.000229 | 0.002324 | 0.000306 | 0.002462 | 0.000261 | 0.033 |  |
| Cerebellar Vermal Lobules, VI-VIII | 0.000990 | 0.000100 | 0.001045 | 0.000111 | 0.000964 | 0.000116 | 0.001075 | 0.000118 | 0.000988 | 0.000121 | 0.001055 | 0.000099 | 0.000* |  |
| Cerebellar Vermal Lobules VIII-XI | 0.001231 | 0.000166 | 0.001242 | 0.000203 | 0.001177 | 0.000256 | 0.001283 | 0.000135 | 0.001232 | 0.000191 | 0.001294 | 0.000160 | 0.080 |  |
| Basal Cerebrum and Forebrain | 0.000935 | 0.000127 | 0.000965 | 0.000101 | 0.000940 | 0.000119 | 0.000978 | 0.000094 | 0.000963 | 0.000092 | 0.000996 | 0.000099 | 0.077 |  |
| Anterior Cingulate Gyri | 0.005880 | 0.000922 | 0.006016 | 0.000859 | 0.005893 | 0.000782 | 0.006299 | 0.000615 | 0.006242 | 0.000863 | 0.006528 | 0.000714 | 0.001* |  |
| Anterior Insula | 0.006142 | 0.000846 | 0.006426 | 0.000659 | 0.006134 | 0.000587 | 0.006332 | 0.000579 | 0.006374 | 0.000620 | 0.006606 | 0.000649 | 0.009* |  |
| Anterior Orbital Gyri | 0.002494 | 0.000290 | 0.002502 | 0.000321 | 0.002474 | 0.000238 | 0.002531 | 0.000227 | 0.002505 | 0.000264 | 0.002588 | 0.000256 | 0.364 |  |
| Angular Gyri | 0.012265 | 0.001707 | 0.012688 | 0.001562 | 0.012279 | 0.001260 | 0.012735 | 0.001153 | 0.012624 | 0.001269 | 0.012980 | 0.001288 | 0.133 |  |
| Calcarine Cortex | 0.004517 | 0.000682 | 0.004610 | 0.000523 | 0.004464 | 0.000686 | 0.004538 | 0.000557 | 0.004580 | 0.000737 | 0.004755 | 0.000873 | 0.485 |  |
| Central Operculum | 0.005287 | 0.000716 | 0.005411 | 0.000483 | 0.005338 | 0.000646 | 0.005279 | 0.000708 | 0.005397 | 0.000539 | 0.005579 | 0.000626 | 0.222 |  |
| Cuneus | 0.005355 | 0.000719 | 0.005480 | 0.000681 | 0.005362 | 0.000648 | 0.005449 | 0.000611 | 0.005671 | 0.000702 | 0.005744 | 0.000578 | 0.022 |  |
| Entorhinal Area | 0.002821 | 0.000276 | 0.002782 | 0.000259 | 0.002923 | 0.000237 | 0.002913 | 0.000277 | 0.002943 | 0.000243 | 0.002939 | 0.000219 | 0.051 |  |
| Frontal Operculum | 0.002609 | 0.000362 | 0.002730 | 0.000320 | 0.002659 | 0.000308 | 0.002703 | 0.000371 | 0.002692 | 0.000306 | 0.002756 | 0.000373 | 0.521 |  |
| Frontal Pole | 0.004046 | 0.000433 | 0.004083 | 0.000361 | 0.004048 | 0.000360 | 0.004104 | 0.000360 | 0.004144 | 0.000426 | 0.004262 | 0.000355 | 0.082 |  |
| Fusiform Gyri | 0.009905 | 0.001127 | 0.010026 | 0.000970 | 0.010199 | 0.001090 | 0.010045 | 0.000615 | 0.010516 | 0.000933 | 0.010499 | 0.000928 | 0.017 |  |
| Rectus Gyri | 0.002362 | 0.000269 | 0.002388 | 0.000258 | 0.002330 | 0.000239 | 0.002368 | 0.000257 | 0.002389 | 0.000256 | 0.002474 | 0.000256 | 0.132 |  |
| Inferior Occipital Gyri | 0.007820 | 0.001126 | 0.007977 | 0.000777 | 0.007889 | 0.000825 | 0.007846 | 0.000735 | 0.008112 | 0.000776 | 0.008343 | 0.000785 | 0.031 |  |
| Inferior Temporal Gyri | 0.013118 | 0.001372 | 0.013768 | 0.001146 | 0.013595 | 0.001461 | 0.013130 | 0.001008 | 0.013899 | 0.001160 | 0.013681 | 0.001208 | 0.037 |  |
| Lingual Gyri | 0.009440 | 0.001086 | 0.009306 | 0.000912 | 0.009465 | 0.000909 | 0.009366 | 0.000672 | 0.009747 | 0.000915 | 0.009961 | 0.000773 | 0.006* |  |
| Lateral Orbital Gyri | 0.002677 | 0.000324 | 0.002699 | 0.000365 | 0.002664 | 0.000275 | 0.002727 | 0.000344 | 0.002794 | 0.000288 | 0.002843 | 0.000294 | 0.043 |  |
| Middle Cingulate Gyri | 0.005886 | 0.000884 | 0.006073 | 0.000607 | 0.005796 | 0.000660 | 0.006228 | 0.000711 | 0.006048 | 0.000710 | 0.006430 | 0.000614 | 0.000* |  |
| Medial Frontal Cortex | 0.002496 | 0.000418 | 0.002518 | 0.000401 | 0.002479 | 0.000347 | 0.002511 | 0.000340 | 0.002532 | 0.000340 | 0.002594 | 0.000343 | 0.714 |  |
| Middle Frontal Gyri | 0.023676 | 0.002974 | 0.023790 | 0.002291 | 0.023609 | 0.002573 | 0.023994 | 0.002246 | 0.024555 | 0.002556 | 0.025284 | 0.002556 | 0.015 |  |
| Middle Occipital Gyri | 0.006564 | 0.001043 | 0.006674 | 0.000734 | 0.006550 | 0.000712 | 0.006778 | 0.000727 | 0.006862 | 0.000723 | 0.006981 | 0.000665 | 0.057 |  |
| Medial Orbital Gyri | 0.005159 | 0.000530 | 0.005270 | 0.000526 | 0.005128 | 0.000439 | 0.005254 | 0.000435 | 0.005208 | 0.000521 | 0.005475 | 0.000499 | 0.012* |  |
| Medial Posterior Cingulate Gyri | 0.001229 | 0.000209 | 0.001285 | 0.000190 | 0.001232 | 0.000214 | 0.001271 | 0.000133 | 0.001251 | 0.000166 | 0.001323 | 0.000152 | 0.103 |  |
| Medial Precentral Gyri | 0.003205 | 0.000434 | 0.003357 | 0.000366 | 0.003157 | 0.000438 | 0.003296 | 0.000364 | 0.003321 | 0.000367 | 0.003417 | 0.000333 | 0.026 |  |
| Superior Medial Frontal Gyri | 0.008890 | 0.001215 | 0.009247 | 0.001080 | 0.008893 | 0.001044 | 0.009204 | 0.000773 | 0.009294 | 0.001011 | 0.009632 | 0.001100 | 0.011* |  |
| Middle Temporal Gyri | 0.017509 | 0.002326 | 0.018569 | 0.001833 | 0.017666 | 0.001931 | 0.017841 | 0.001829 | 0.018352 | 0.001682 | 0.018272 | 0.001622 | 0.134 |  |
| Occipital Pole | 0.004101 | 0.000682 | 0.004176 | 0.000549 | 0.004144 | 0.000472 | 0.004180 | 0.000461 | 0.004265 | 0.000536 | 0.004317 | 0.000494 | 0.443 |  |
| Occipital Fusiform Gyri | 0.004206 | 0.000686 | 0.004186 | 0.000482 | 0.004276 | 0.000520 | 0.004074 | 0.000347 | 0.004340 | 0.000458 | 0.004384 | 0.000427 | 0.094 |  |
| Inferior Frontal Gyri | 0.004470 | 0.000730 | 0.004642 | 0.000433 | 0.004390 | 0.000518 | 0.004581 | 0.000468 | 0.004482 | 0.000526 | 0.004709 | 0.000516 | 0.064 |  |
| Inferior Frontal Orbital Gyri | 0.001911 | 0.000289 | 0.001933 | 0.000307 | 0.001908 | 0.000236 | 0.001972 | 0.000295 | 0.001987 | 0.000196 | 0.001988 | 0.000234 | 0.478 |  |
| Posterior Cingulate Gyri | 0.005520 | 0.000694 | 0.005607 | 0.000606 | 0.005595 | 0.000604 | 0.005637 | 0.000575 | 0.005768 | 0.000495 | 0.005741 | 0.000603 | 0.379 |  |
| Precuneus | 0.013755 | 0.001712 | 0.013897 | 0.001466 | 0.013761 | 0.001513 | 0.013948 | 0.001107 | 0.014444 | 0.001281 | 0.014494 | 0.001259 | 0.026 |  |
| Parahippocampal Gyri | 0.003782 | 0.000301 | 0.003876 | 0.000327 | 0.003916 | 0.000315 | 0.003997 | 0.000244 | 0.003961 | 0.000297 | 0.004049 | 0.000321 | 0.006* |  |
| Posterior Insula | 0.003119 | 0.000494 | 0.003158 | 0.000296 | 0.003075 | 0.000270 | 0.003190 | 0.000319 | 0.003202 | 0.000314 | 0.003319 | 0.000349 | 0.020 |  |
| Parietal Operculum | 0.003036 | 0.000494 | 0.002918 | 0.000373 | 0.002962 | 0.000479 | 0.002960 | 0.000517 | 0.003077 | 0.000442 | 0.003175 | 0.000524 | 0.170 |  |
| Postcentral Gyri | 0.011780 | 0.001237 | 0.012398 | 0.001490 | 0.011902 | 0.001321 | 0.012211 | 0.001062 | 0.012012 | 0.000926 | 0.012951 | 0.001315 | 0.000* |  |
| Posterior Orbital Gyri | 0.003501 | 0.000555 | 0.003487 | 0.000460 | 0.003537 | 0.000410 | 0.003542 | 0.000346 | 0.003718 | 0.000427 | 0.003756 | 0.000453 | 0.016 |  |
| Planum Polare | 0.002652 | 0.000429 | 0.002694 | 0.000278 | 0.002593 | 0.000234 | 0.002677 | 0.000290 | 0.002723 | 0.000306 | 0.002843 | 0.000345 | 0.008* |  |
| Precentral Gyri | 0.015637 | 0.001496 | 0.016136 | 0.001354 | 0.015477 | 0.001660 | 0.015641 | 0.001133 | 0.015864 | 0.001299 | 0.016577 | 0.001381 | 0.003* |  |
| Planum Temporale | 0.002753 | 0.000404 | 0.002720 | 0.000294 | 0.002635 | 0.000394 | 0.002648 | 0.000322 | 0.002799 | 0.000370 | 0.002823 | 0.000431 | 0.144 |  |
| Subcallosal Area | 0.001523 | 0.000227 | 0.001570 | 0.000214 | 0.001519 | 0.000201 | 0.001498 | 0.000164 | 0.001588 | 0.000196 | 0.001569 | 0.000208 | 0.311 |  |
| Superior Frontal Gyri | 0.017984 | 0.002098 | 0.018186 | 0.001690 | 0.017860 | 0.001711 | 0.017813 | 0.001222 | 0.018510 | 0.001618 | 0.019237 | 0.001456 | 0.000* |  |
| Supplementary Motor Cortex | 0.006889 | 0.000763 | 0.007019 | 0.000862 | 0.006792 | 0.000757 | 0.007340 | 0.000687 | 0.007266 | 0.000609 | 0.007396 | 0.000786 | 0.001* |  |
| Supramarginal Gyri | 0.009829 | 0.001409 | 0.010006 | 0.000988 | 0.009789 | 0.001048 | 0.009934 | 0.000900 | 0.009980 | 0.001037 | 0.010475 | 0.001245 | 0.042 |  |
| Superior Occipital Gyri | 0.004227 | 0.000586 | 0.004416 | 0.000486 | 0.004401 | 0.000530 | 0.004553 | 0.000444 | 0.004413 | 0.000517 | 0.004632 | 0.000499 | 0.018 |  |
| Superior Parietal Lobule | 0.011894 | 0.001537 | 0.012665 | 0.001459 | 0.011923 | 0.001188 | 0.012564 | 0.000931 | 0.012348 | 0.001012 | 0.012765 | 0.001114 | 0.003* |  |
| Superior Temporal Gyri | 0.008536 | 0.001112 | 0.008826 | 0.001048 | 0.008467 | 0.000754 | 0.008676 | 0.000803 | 0.008847 | 0.000803 | 0.008928 | 0.000894 | 0.123 |  |
| Temporal Pole | 0.010417 | 0.001054 | 0.010840 | 0.001121 | 0.010676 | 0.000859 | 0.010752 | 0.000998 | 0.010973 | 0.000885 | 0.010877 | 0.001033 | 0.208 |  |
| Inferior Frontal Angular Gyri | 0.004283 | 0.000636 | 0.004410 | 0.000547 | 0.004231 | 0.000586 | 0.004391 | 0.000450 | 0.004319 | 0.000417 | 0.004548 | 0.000476 | 0.045 |  |
| Temporal Transverse Gyri | 0.001747 | 0.000306 | 0.001714 | 0.000232 | 0.001710 | 0.000236 | 0.001705 | 0.000216 | 0.001780 | 0.000227 | 0.001812 | 0.000289 | 0.301 |  |

Note. The displayed *p*-values are not corrected for multiple comparisons. *Regions surviving *p* < 0.05 after false discovery rate (FDR) correction.

Supplementary Table e-3. Standardized canonical discriminant function coefficients.

**Regions Full model Males-only Females-only**

|  | **Function 1** | **Function 2** | **Function 1** | **Function 2** | **Function 1** | **Function 2** |
| --- | --- | --- | --- | --- | --- | --- |
| Accumbens | 0.13 | 0.38 | -0.03 | -0.58 | 0.38 | -0.19 |
| Amygdala | -0.41 | -0.07 | -0.93 | -0.64 | -0.86 | -1.19 |
| Caudate | 0.14 | 0.33 | -0.04 | -0.57 | -0.24 | 0.72 |
| Exterior Cerebellum | -0.40 | -0.16 | -0.27 | 0.47 | -0.76 | 0.50 |
| Hippocampus | 0.55 | 0.30 | 0.91 | 0.38 | 0.98 | 1.10 |
| Pallidum | 0.10 | -0.04 | 0.25 | 0.06 | -0.31 | 0.18 |
| Putamen | -0.27 | -0.41 | 0.51 | 0.44 | 0.04 | 0.69 |
| Thalamus | 0.41 | -0.15 | 0.63 | -0.54 | 0.12 | -0.55 |
| Cerebellar Vermal Lobules, I-V | -0.26 | 0.23 | -0.39 | -0.08 | 0.18 | 0.05 |
| Cerebellar Vermal Lobules, VI-VIII | -0.12 | -0.15 | -0.13 | 0.43 | 0.02 | 0.43 |
| Cerebellar Vermal Lobules VIII-XI | 0.31 | -0.16 | 0.44 | -0.06 | 0.43 | -0.41 |
| Basal Cerebrum and Forebrain | -0.50 | 0.29 | -1.32 | -0.03 | -0.92 | -0.62 |
| Anterior Cingulate Gyri | 0.31 | 0.22 | 0.46 | -0.50 | 0.25 | -0.02 |
| Anterior Insula | 0.08 | -0.61 | 0.38 | 0.65 | 0.27 | -0.95 |
| Anterior Orbital Gyri | -0.52 | 0.00 | -0.57 | 0.06 | -0.18 | 0.80 |
| Angular Gyri | -0.18 | 0.52 | -0.52 | -0.41 | 0.32 | 0.10 |
| Calcarine Cortex | -0.23 | -0.25 | -0.63 | 0.55 | -0.34 | -0.67 |
| Central Operculum | -0.29 | -0.02 | -0.15 | -0.19 | -0.06 | -0.17 |
| Cuneus | -0.05 | -0.52 | 0.52 | 0.53 | -1.10 | -0.32 |
| Entorhinal Area | 0.32 | 0.50 | -0.03 | 0.07 | 1.03 | 0.53 |
| Frontal Operculum | 0.12 | 0.71 | -0.41 | -0.92 | -0.10 | 0.29 |
| Frontal Pole | 0.10 | -0.06 | 0.22 | -0.04 | 0.34 | -0.32 |
| Fusiform Gyri | 0.54 | 0.37 | 0.68 | -0.61 | 1.22 | 0.13 |
| Rectus Gyri | 0.35 | -0.34 | 0.77 | -0.29 | 0.29 | -0.24 |
| Inferior Occipital Gyri | 0.31 | -0.06 | 1.07 | -0.40 | 0.75 | -0.15 |
| Inferior Temporal Gyri | -0.32 | 0.38 | -0.75 | -0.72 | -1.39 | 0.19 |
| Lingual Gyri | 0.56 | -0.30 | -0.03 | 0.17 | 1.06 | -0.58 |
| Lateral Orbital Gyri | 0.25 | 0.10 | 0.63 | 0.08 | 0.02 | 0.25 |
| Middle Cingulate Gyri | -0.31 | -0.19 | -0.23 | 0.36 | 0.44 | 0.03 |
| Medial Frontal Cortex | -0.29 | 0.43 | -0.55 | 0.01 | 0.30 | 0.35 |
| Middle Frontal Gyri | 0.99 | 0.10 | 0.98 | -0.67 | 0.03 | 0.77 |
| Middle Occipital Gyri | 0.26 | -0.37 | -0.24 | 0.87 | 0.16 | -0.53 |

| Medial Orbital Gyri | -0.41 | 0.08 | -1.23 | 1.13 | 0.18 | 0.10 |
| --- | --- | --- | --- | --- | --- | --- |
| Medial Posterior Cingulate Gyri | -0.11 | 0.29 | -0.32 | 0.07 | -0.23 | 0.42 |
| Medial Precentral Gyri | -0.15 | -0.29 | 0.25 | -0.28 | -0.98 | -0.87 |
| Superior Medial Frontal Gyri | -0.08 | -0.09 | 0.26 | -0.58 | -0.54 | -0.86 |
| Middle Temporal Gyri | -0.58 | -0.67 | 0.28 | 1.06 | -1.02 | 0.23 |
| Occipital Pole | -0.20 | 0.35 | 0.30 | -0.10 | 0.20 | 0.86 |
| Occipital Fusiform Gyri | -0.29 | -0.12 | -0.63 | 0.14 | -0.57 | -0.42 |
| Inferior Frontal Gyri | -0.40 | 0.07 | -0.68 | 0.27 | -0.63 | -0.21 |
| Inferior Frontal Orbital Gyri | -0.16 | -0.07 | 0.39 | 0.32 | 0.19 | 0.52 |
| Posterior Cingulate Gyri | -0.29 | 0.07 | 0.36 | 0.61 | -1.44 | 0.00 |
| Precuneus | 0.57 | 0.21 | 0.00 | -0.70 | 1.33 | 0.40 |
| Parahippocampal Gyri | -0.06 | 0.05 | -0.28 | -0.27 | 0.33 | 0.28 |
| Posterior Insula | 0.17 | -0.05 | -0.02 | -0.02 | 0.03 | 0.05 |
| Parietal Operculum | 0.02 | 0.58 | -0.24 | -0.43 | 1.77 | 0.53 |
| Postcentral Gyri | 0.32 | -0.09 | 0.63 | -0.29 | -0.13 | -0.06 |
| Posterior Orbital Gyri | 0.86 | -0.12 | 1.18 | -0.95 | 0.43 | -0.38 |
| Planum Polare | -0.03 | -0.13 | -0.78 | 0.00 | 0.02 | -0.33 |
| Precentral Gyri | -0.38 | -0.71 | -0.39 | 0.60 | 0.07 | -0.44 |
| Planum Temporale | -0.04 | -0.85 | 0.77 | 0.57 | -2.06 | -1.17 |
| Subcallosal Area | 0.06 | -0.33 | 0.35 | 0.19 | -0.24 | 0.42 |
| Superior Frontal Gyri | 0.40 | -0.30 | -0.26 | -0.37 | -0.33 | -1.25 |
| Supplementary Motor Cortex | 0.31 | 0.27 | 0.39 | 1.29 | 1.80 | 1.36 |
| Supramarginal Gyri | 0.12 | -0.24 | -0.23 | 0.53 | 0.10 | -0.15 |
| Superior Occipital Gyri | 0.18 | 0.99 | -0.15 | -1.00 | 0.53 | 0.48 |
| Superior Parietal Lobule | -0.33 | -0.25 | 0.37 | 0.31 | -0.43 | -0.05 |
| Superior Temporal Gyri | -0.23 | 0.19 | -0.13 | -0.36 | 0.07 | -0.24 |
| Temporal Pole | 0.28 | -0.10 | 0.94 | -0.09 | -0.24 | 0.00 |
| Inferior Frontal Angular Gyri | -0.49 | 0.15 | -0.79 | 0.57 | 0.27 | 0.69 |
| Temporal Transverse Gyri | -0.23 | 0.16 | -0.72 | 0.07 | -0.03 | 0.60 |

Note. The full model includes all bilaterally combined regions among males and females.

Supplementary Table e-4. Confusion matrix of the full model.

| **Full Model** | | **Predicted Group Membership** | | |  |
| --- | --- | --- | --- | --- | --- |
| **Original** | **Group** | **Unimpaired PWH** | **HAND** | **Uninfected Control** | **Total** |
|  | **Unimpaired PWH** | 33 | 8 | 21 | 62 |
|  | **HAND** | 3 | 34 | 11 | 48 |
|  | **Uninfected Control** | 17 | 5 | 99 | 121 |
|  | **Total** | 53 | 47 | 131 | 231 |

Supplementary Table e-5. Confusion matrix of the male model.

| **Male Model** | | **Predicted Group Membership** | | |  |
| --- | --- | --- | --- | --- | --- |
| **Original** | **Group** | **Unimpaired PWH** | **HAND** | **Uninfected Control** | **Total** |
|  | **Unimpaired PWH** | 30 | 2 | 5 | 37 |
|  | **HAND** | 3 | 23 | 1 | 27 |
|  | **Uninfected Control** | 4 | 1 | 59 | 64 |
|  | **Total** | 37 | 26 | 65 | 128 |

Supplementary Table e-6. Confusion matrix of the female model.

| **Female Model** | | **Predicted Group Membership** | | |  |
| --- | --- | --- | --- | --- | --- |
| **Original** | **Group** | **Unimpaired PWH** | **HAND** | **Uninfected Control** | **Total** |
|  | **Unimpaired PWH** | 23 | 0 | 2 | 25 |
|  | **HAND** | 0 | 21 | 0 | 21 |
|  | **Uninfected Control** | 1 | 1 | 55 | 57 |
|  | **Total** | 24 | 22 | 57 | 103 |

Supplementary Table e-7. Confusion matrix of the overall model stratified by sex.

| **Overall Model Stratified by Sex** | | **Predicted Group Membership** | | |  |
| --- | --- | --- | --- | --- | --- |
| **Original** | **Group** | **Unimpaired PWH** | **HAND** | **Uninfected Control** | **Total** |
|  | **Unimpaired PWH** | 53 | 2 | 7 | 62 |
|  | **HAND** | 3 | 44 | 1 | 48 |
|  | **Uninfected Control** | 5 | 2 | 114 | 121 |
|  | **Total** | 61 | 48 | 122 | 231 |
